# Current Treatment of Toxoplasma Retinochoroiditis: An Evidence-Based Review

**DOI:** 10.1155/2014/273506

**Published:** 2014-08-13

**Authors:** Meredith Harrell, Petros E. Carvounis

**Affiliations:** ^1^Texas Tech University, Health Sciences Center, Paul L. Foster School of Medicine, El Paso, TX 79905, USA; ^2^Cullen Eye Institute, Baylor College of Medicine, 1977 Butler Boulevard, Houston, TX 77030, USA

## Abstract

*Objective*. To perform an evidence-based review of treatments for *Toxoplasma* retinochoroiditis (TRC). *Methods*. A systematic literature search was performed using the PubMed database and the key phrase “ocular toxoplasmosis treatment” and the filter for “controlled clinical trial” and “randomized clinical trial” as well as OVID medline (1946 to May week 2 2014) using the keyword ‘‘ocular toxoplasmosis”. The included studies were used to evaluate the various treatment modalities of TRC. *Results*. The electronic search yielded a total of 974 publications of which 44 reported on the treatment of ocular toxoplasmosis. There were 9 randomized controlled studies and an additional 3 comparative studies on the treatment of acute TRC with systemic or intravitreous antibiotics or on reducing the recurrences of TRC. Endpoints of studies included visual acuity improvement, inflammatory response, lesion size changes, recurrences of lesions, and adverse effects of medications. *Conclusions*. There was conflicting evidence as to the effectiveness of systemic antibiotics for TRC. There is no evidence to support that one antibiotic regimen is superior to another so choice needs to be informed by the safety profile. Intravitreous clindamycin with dexamethasone seems to be as effective as systemic treatments. There is currently level I evidence that intermittent trimethoprim-sulfamethoxazole prevents recurrence of the disease.

## 1. Introduction

Ocular toxoplasmosis is the commonest cause of posterior uveitis and is usually the result of an acquired infection caused by the protozoan* Toxoplasma gondii* [1, 2]. The most common manifestation of ocular toxoplasmosis is* Toxoplasma* retinochoroiditis which is typically a unilateral, unifocal, large lesion (greater than 1 disc diameter) typically associated with vitreitis that is in the posterior pole in two-thirds of cases [2, 3]. A granulomatous anterior chamber inflammation is frequent, and retina vasculitis (usually arteriolitis) is present in about a third of patients [2–5]. Visual acuity loss during acute toxoplasma retinochoroiditis results from vitreitis or from involvement of the macula or optic nerve. Visual loss may become permanent due to formation of a macular scar or due to optic atrophy so that 24% of patients have vision of 20/200 or less in at least one eye [5, 6]. The scarring resulting from Toxoplasma retinochoroiditis can be associated with severe visual field loss when it occurs close to the optic disc [7].

There is no consensus as to what the best treatment for* Toxoplasma* retinochoroiditis might be. The most recent systematic evidence-based review of the literature considered articles published up to July 2011 [8]. There have been significant additional contributions to the literature since that time and we wished to repeat a systematic evidence-based review of the literature incorporating our observations on the studies reviewed. We therefore performed this updated systematic literature review to evaluate the treatments for toxoplasma retinochoroiditis.

## 2. Literature Search

A PubMed (National Library of Medicine) search was conducted using the key phrase “ocular toxoplasmosis treatment” and a filter for “controlled clinical trial” and “randomized clinical trial.” Additionally an OVID medline (1946-May week 2 2014) search was conducted using the keyword “ocular toxoplasmosis.” Articles were limited to articles published in English. There were no restrictions on age, ethnicity, or geographic locations of patients.

## 3. Results

We found a total of 974 publications and reviewed the abstracts to select publications reporting on treatment outcomes of* Toxoplasma* retinochoroiditis. We found 29 publications written in English reporting on outcomes of treatment of* Toxoplasma *retinochoroiditis. The studies used various combinations of endpoints to determine the efficacy and safety of the medications. All studies reported improvement in symptoms associated with ocular toxoplasmosis after treatment. Resolution or improvement in ocular findings was seen within varying time points ranging from 6 weeks to 20 months between trials. There were sources of clinical heterogeneity among studies such as duration and severity of ocular toxoplasmosis, age, and previous treatments used by patients. Therapies also varied in their dosages, duration, frequency, and combinations, making it difficult to compare across studies. There were several studies where the scales used for evaluating endpoint parameters were not well-defined and quality of life and subjective assessments of treatments were not found among the reviewed studies.

We used three subheadings to discuss the treatments of* Toxoplasma* retinochoroiditis: systemic antibiotic treatments, intravitreous antibiotic treatments, and treatments to reduce the rate of recurrence of toxoplasma retinochoroiditis.

### 3.1. Systemic Antibiotic Treatments for Active* Toxoplasma* Retinochoroiditis

In 1956, Perkins and colleagues published a double-masked, randomized, and controlled study which included 43 patients with* Toxoplasma *retinochoroiditis treated using either a 2-week course of pyrimethamine or placebo, showing statistically significant improvement compared to placebo [9]. Since that time a number of mainly noncomparative case series have been published purporting that clindamycin [10–12], spiramycin [13], azithromycin [14, 15], trimethoprim-sulfamethoxazole [16], atovaquone [17], alone or in combination with pyrimethamine, and/or sulfadiazine are effective in the treatment of toxoplasmosis. Given the self-limiting nature of* Toxoplasma* retinochoroiditis in immunocompetent individuals noncomparative case series have little role in establishing the efficacy of any particular agent, especially compared to established treatments. We found 2 retrospective comparative studies, 2 prospective comparative studies (although there was significant overlap of patients reported in these 2 studies), and 4 randomized controlled studies on the systemic treatment of* Toxoplasma gondii* retinochoroiditis.

#### 3.1.1. Prospective or Retrospective Comparative Studies

A retrospective, comparative, single-centre study published in 1962 by Fajardo et al. [18] compared the efficacy of 3 treatment regimens for* Toxoplasma* retinochoroiditis on 87 patients. The treatments consisted of pyrimethamine (100 mg initially, then 50 mg), sulfadiazine (1 g qid), and methylprednisolone (4 mg tid); spiramycin (2 g qd) and methylprednisolone (4 mg tid); and methylprednisolone (4 mg tid) alone. The authors reported that the interval to inactivity (resolution of inflammation and scarring of the retinal lesion) was shorter in the group treated with pyrimethamine and sulfadiazine with a statistically significant greater proportion of patients becoming inactive within the first 8 weeks compared to the other treatments, with no differences in visual outcomes [18].

Similarly in a retrospective, comparative, and single-centre study published by Nolan and Rosen reporting on 69 patients, the efficacy of 2 treatments for Toxoplasma retinochoroiditis was compared to treatment with corticosteroids or observation [19]. The treatments were either pyrimethamine (100 mg loading dose then 25 mg daily) or spiramycin (1–4 g daily). Pyrimethamine, but not spiramycin, was found to have significantly reduced the healing time [19].

The above results were in contrast to the initial report from a prospective multicenter study from the Netherlands that compared 3 treatment regimens to observation [20]. The treatment regimens consisted of either pyrimethamine (100 mg for 1 day, then 25 mg bid), sulfadiazine (1 g qid), folinic acid (5 mg), and prednisone (60 mg then taper); clindamycin (300 mg qid), sulfadiazine (1 g qid), and prednisone (60 mg, then taper); or trimethoprim-sulfamethoxazole (160–800 mg bid for 2 weeks then 80–400 mg bid). The 106 patients recruited were assigned to treatment depending on the center at which they were treated (not randomly); they were assigned to observation if the lesions were in the periphery. The authors reported no significant differences between treatments or comparing the treatments to observation in terms of duration of inflammatory activity or reduction in size of the lesion. Visual outcomes or rates of recurrence were not reported. The pyrimethamine-sulfadiazine group had the highest frequency of adverse events (52%), including thrombocytopenia, leukopenia, rashes, and fever [20].

The same group from the Netherlands then published an overlapping publication with 149 patients assigned to the groups described above (presumably the 106 patients in their prior publication were included) [21]. Again there was no difference in the duration of inflammatory activity, visual acuity, or rate of recurrence (mean 49% at 3 years) between the treated and untreated groups. The authors reported that there was marked decrease (at least 0.5 disc diameter) in the size of the lesion in 49% of pyrimethamine treated patients compared to 28% in clindamycin-treated patients, 11% of trimethoprim-sulfamethoxazole treated patients, and 20% in the observation group. The difference between the pyrimethamine group and the observation group was statistically significant for this measure. It should be noted that the lesion size was measured from fundus photographs in the treatment groups (as the lesion was in the posterior pole) while for lesions in the observation groups the lesion size was estimated from drawings of the peripheral retina; the comparison may, therefore, have been biased to show greater efficacy in the treatment groups. Moreover, a chi-squared test was used with no attempt to adjust for multiple comparisons. Further, the original publication in 1989 had found no statistically significant difference and it was only when the additional 33 patients were added that such a difference was found in the 1993 paper by the same group [21]. It is therefore unfortunate that subsequent reviews of the literature on the treatment of ocular toxoplasmosis have given much weight to this finding. In our view, these overlapping papers support the use of observation for peripheral lesions and suggest that all the treatments employed in the study had similar efficacy with pyrimethamine-sulfadiazine having the worse systemic safety profile.

#### 3.1.2. Randomized-Controlled Studies of Oral Antimicrobials for Active* Toxoplasma* Retinochoroiditis

*Triple Therapy versus Steroid Alone*. A randomized, placebo-controlled, and double-masked study by Acers [22] compared the efficacy of pyrimethamine (200 mg on day 1, 100 mg on day 2, 50 mg on days 3–15, and 25 mg on days 16–56), trisulfapyrimidines (2 g), and prednisone 40 mg to prednisone 40 mg alone for active toxoplasma retinochoroiditis. Only 20 patients were recruited to the study and randomized 1 : 1 to each of the groups. No difference was found in the time to inactivity or visual acuity between the 2 groups. In the pyrimethamine-trisulfapyrimidine group 30% of patients developed an adverse event (usually nausea, anorexia, or arthralgia), with 1 patient developing severe thrombocytopenia [22]. The study was limited by the low patient numbers. While the study further questions the efficacy of routine systemic antibiotics for* Toxoplasma* retinochoroiditis it cannot be overstated that several studies since have documented that corticosteroid administration without antiparasitic treatment can lead to a fulminant necrotizing retinochoroiditis and worse visual outcomes [6].

*Trimethoprim-Sulfamethoxazole versus Triple Therapy*. A randomized, single-blinded study by Soheilian et al. [23] on 59 patients compared the efficacy and safety of trimethoprim-sulfamethoxazole (160 mg–800 mg) against classic therapy triple therapy with pyrimethamine (100 mg for 2 days, then 25 mg daily), sulfadiazine (2 g), and folinic acid (5 mg) with both treatment groups receiving adjuvant prednisone. Randomization was 1 : 1. No significant differences were found in terms of lesion size, mean improvement in visual acuity, recurrence rates, and adverse events to drug therapy, although 5 patients (17%) in each group were lost to follow-up. One patient in each treatment group developed an adverse reaction to their respective treatment (both developed a rash). The authors concluded that trimethoprim-sulfamethoxazole was a reasonable alternative to classic triple therapy; [23] however, the study has been criticized for using half the dose of pyrimethamine and sulfadiazine commonly used in clinical practice, as well as the large numbers of patients lost to follow-up and limited numbers of patients recruited to the study.

*Azithromycin versus Triple Therapy*. Two studies compared regimens with azithromycin against triple therapy with pyrimethamine, sulfadiazine, and folinic acid. In a 2002 randomized, open-label, and controlled study, Bosch-Driessen et al. [24] compared the efficacy of 4 weeks of azithromycin (250 mg)-pyrimethamine (100 mg on day 1, then 50 mg)-folinic acid (15 mg) versus sulfadiazine (4 g)-pyrimethamine (100 mg on day 1, then 50 mg)-folinic acid (15 mg), or the treatment of active toxoplasma retinochoroiditis. Randomization of the 43 patients was 1 : 1. Both groups received adjuvant prednisone. There were no significant differences between treatment groups on the duration of inflammation, change in lesion size, improvement in visual acuity, or risk of recurrence. Adverse effects were more frequent in the sulfadiazine group (64%), requiring discontinuation of treatment in 3 patients (14%). Adverse effects were less common in the azithromycin group (33%), although 1 patient in the azithromycin group died from a cerebral aneurysm during the course of the study. The study provides some evidence that azithromycin with pyrimethamine and folinic acid is a reasonable alternative to sulfadiazine with pyrimethamine and folinic acid [24].

In a more recent randomized, open-label study, Balaskas et al. [25] compared azithromycin (500 mg) to triple therapy consisting of 50 mg pyrimethamine, 4 g of sulfadiazine (3 g if the patient weighed less than 65 kg), and folinic acid (15 mg); both groups received adjuvant prednisone. Patients were randomized 1 : 1 to each of the groups. There was no significant difference in the number of responders to treatment, with all the patients responding to treatment in the triple therapy group and 90% of patients responding to treatment in the azithromycin group. Adverse events such as malaise, dizziness, headaches, and gastrointestinal disturbances were reported by all patients in the triple therapy group compared to none in the azithromycin group. The study was limited by small numbers, having recruited a total of 19 patients [25]. Therefore the question of whether azithromycin is or not as effective as triple therapy remains unanswered, although it appears that it is better tolerated than triple therapy.

There is conflicting evidence as to whether systemic antibiotics are effective in the treatment of toxoplasma retinochoroiditis in the first place, although the preponderance of evidence suggests some effects [9, 18–22]. Pyrimethamine is known to frequently result in bone marrow suppression leading to thrombocytopenia, leukopenia, and anemia [9, 26], while severe hepatotoxicity is a well-known complication of sulfadiazine therapy [26, 27], skin rashes, anorexia, nausea, and lassitude are quite common with either medication [25]. There is some evidence [20, 21, 23–25], including that from randomized clinical trials [23–25], to suggest that trimethoprim-sulfamethoxazole or azithromycin may be no less effective than pyrimethamine-sulfadiazine and both of the former have more adverse effects than the latter. There is also some evidence from a prospective comparative trial to suggest that this may also be true of clindamycin, although the systemic safety profile for clindamycin (mainly gastrointestinal upset) is worse than that of trimethoprim-sulfamethoxazole or azithromycin [20, 21]. Interestingly, a recent meta-analysis of treatment of toxoplasmic encephalitis in HIV-infected patients showed that trimethoprim-sulfamethoxazole was noninferior to pyrimethamine-sulfadiazine [28]. Trimethoprim-sulfamethoxazole is readily available and is the least expensive of the two so may be the best first-line treatment if the clinician is inclined not to observe* Toxoplasma* retinochoroiditis.

### 3.2. Intravitreous Treatments for* Toxoplasma* Retinochoroiditis

Tabbara and colleagues demonstrated the efficacy of periocular clindamycin (subTenon's or retrobulbar) in a rabbit model of* Toxoplasma* retinochoroiditis in the 1970s [29, 30]. Dr. Peyman's group then reported the resolution of inflammation and improvement in the vision following intravitreous clindamycin and dexamethasone (IVTCD) together with systemic sulfadiazine in the first trimester of pregnancy of a patient with a* Toxoplasma* retinochoroiditis lesion in the maculopapillary bundle [31]. Two noncomparative retrospective case series described 6 and 12 patients, respectively, with* Toxoplasma* retinochoroiditis that were treated with IVTCD due to intolerance, contraindication (pregnancy), or lack of response to oral medication and both reported functional and anatomic improvement [32, 33]. Given the generally self-limiting nature of toxoplasma retinochoroiditis case series such as the above do not establish the efficacy of intravitreous treatment for this condition. We found 2 randomized clinical trials evaluating intravitreous clindamycin-dexamethasone for* Toxoplasma* retinochoroiditis [34].

#### 3.2.1. Randomized, Controlled Studies Evaluating Intravitreous Clindamycin-Dexamethasone for* Toxoplasma* Retinochoroiditis

In a randomized, single-masked trial, Sohleilianet al. [34] investigated the efficacy of intravitreous clindamycin (1 mg) and dexamethasone (0.4 mg) compared to pyrimethamine (75 mg for 2 days, then 25 mg), sulfadiazine (4 g for 2 days, then 2 g daily), folinic acid (5 mg), and prednisone. The 81 patients participating in the study were randomized 1 : 1 to each group and follow-up was available in 84% of patients. In the IVTCD group, 47% of patients required more than one injection; IVTCD could be repeated every 2 weeks based on clinical response at the discretion of the examiner. No significant differences between the two groups in the primary endpoint of lesion size reduction were reported; similarly the authors found no differences between the two groups in improvement in visual acuity, resolution of the vitreous inflammation, or rates of recurrence (5.9% in each group by 2 years). There were 2 serious adverse events in the group treated with triple therapy (1 patient developed a severe rash and another thrombocytopenia necessitating cessation of treatment in both cases); in the group receiving IVTCD there were injection-site related complications (subconjunctival hemorrhage) but no systemic adverse events. Of note, the study discovered that IgM-positive cases responded better to classic therapy and IgM-negative cases respond better to IVCD therapy in terms of lesion size reduction [34]. It should be noted that the dose of pyrimethamine and sulfadiazine used in this study was half the dose commonly used in clinical practice in the United States; additionally, there was a 16% loss to follow-up, analysis was not carried out on an intent-to-treat basis, and the numbers were limited, somewhat limiting the clinical applicability of the study's findings.

A randomized, single-masked study by Baharivand and colleagues [35] compared intravitreous clindamycin (1 mg) and dexamethasone (0.4 mg) with pyrimethamine (75 mg for 2 days, then 25 mg), sulfadiazine (2 mg for 2 days, then 4 mg), folinic acid (5 mg), and prednisone (50 mg) for 6 weeks. Sixty-eight patients were randomized 1 : 1 to each treatment group. There was no significant difference between the two groups in terms of change in visual acuity, lesion size, resolution of inflammation, or recurrence rate. In the IVTCD group 88% of patients received a single injection. There was 1 episode of hepatotoxicity reported in the triple therapy group and there were no adverse drug events in the IVCD group. It should be noted that the dose of pyrimethamine used in this study was half that in common clinical practice in the United States [35].

Despite the limitations of the above studies, the preponderance of the (currently limited) evidence suggests that intravitreous clindamycin and dexamethasone is a reasonable alternative to systemic antimicrobial therapy in patients unresponsive or intolerant to oral anti-Toxoplasma medications or when these are contraindicated due to pregnancy. Further, the current evidence, while weak, does not refute the opinion that it is not unreasonable to use IVTCD as a first line treatment. It should be noted that a significant proportion of patients need IVTCD repeated every 1-2 weeks. The greatest advantage of this treatment is its systemic safety profile, although it should be noted that there has been a case report of a generalized rash following intravitreous clindamycin; therefore patients with a known allergy to oral clindamycin may not be suitable candidates for this treatment [36].

### 3.3. Treatments to Reduce Recurrent Rates of* Toxoplasma* Retinochoroiditis

Three approaches have been evaluated to prevent recurrences of toxoplasma retinochoroiditis. The first such approach historically was the application of laser photocoagulation directly on the lesion or in the immediately surrounding retina. For example, in 1966, Spalter et al. [37] presented a case series of 24 patients with a history of recurrent toxoplasma retinochoroiditis whose lesions were surrounded with laser photocoagulation. During a follow-up period ranging from 8 to 33 months there were only 2 recurrences (8%) and these were distant to the lesions treated [37]. However, in a case series of 35 patients that received laser photocoagulation around foci of* Toxoplasma* retinochoroiditis the recurrence rate was 53% in 5 years [38]. Further, in a comparative study of 33 patients treated either with laser around the foci or with triple therapy there was no difference in the rate of recurrence between the two groups [39]. Laser photocoagulation of* Toxoplasma* retinochoroiditis lesions is not a current practice to prevent recurrences given the above evidence.

A second approach was the use of atovaquone or azithromycin to treat acute episodes of* Toxoplasma* retinochoroiditis. Both atovaquone and azithromycin have demonstrated cysticidal activity in preclinical models and it had been hoped that acute treatment with one of these agents would prevent recurrence of toxoplasma retinochoroiditis [40]. Unfortunately, it is clear that this is not the case. The largest series of patients treated with atovaquone was a retrospective case series of 41 patients treated for 6 weeks: the recurrence rate was 27% by 2 years and 75% by 6 years [41]. Similarly, Rothova et al. [15] published a retrospective case series of 11 immunocompetent patients who were treated for toxoplasma retinochoroiditis with a 5-week course of azithromycin; recurrence was noted in 27% of patients within the first year [15]. Further, in a randomized controlled study comparing the combination of azithromycin with pyrimethamine versus sulfadiazine with pyrimethamine there was no statistical difference in the rate of recurrences [24]. Therefore, while atovaquone or azithromycin are reasonable treatment options for treatment of acute* Toxoplasma* retinochoroiditis they have no role in preventing recurrences.

Long-term use of anti-Toxoplasma agents to prevent recurrences has been the third approach evaluated. Indeed, in a prospective, randomized, and open-label trial, Silveira et al. [42] studied the effects of long-term intermittent trimethoprim-sulfamethoxazole (160 mg–800 mg) on recurrence rates of toxoplasmic retinochoroiditis. In this study, 124 patients with a history of recurrent toxoplasma chorioretinitis documented clinically and with positive serology for* T. gondii* were randomized 1 : 1 to an observation group or to receive trimethoprim-sulfamethoxazole every 3 days for 20 consecutive months. The endpoint of recurrence of* Toxoplasma* retinochoroiditis was met by 23.8% of patients in the observation group and 6.6% of patients in the treatment group, a difference that was statistically significant. There were no qualitative differences between recurrences (e.g., amount of inflammation, extent of active retinochoroiditis, etc.) in the 2 groups. It should be noted that 4 patients (15.5%) in the treatment group withdrew from the study due to mild allergic reactions while an additional 2 patients (3.2%) in the treatment group and 4 patients (15.5%) in the control group were lost to follow-up [42].

More recently, Felix et al. [43] published the results of a well-conducted double-masked randomized placebo-controlled study on the effects of trimethoprim-sulfamethoxazole on recurrence rates of toxoplasma retinochoroiditis. In this study, following treatment for active toxoplasma retinochoroiditis with trimethoprim-sulfamethoxazole (160 mg–800 mg) for 45 days, 95 patients were randomized 1 : 1 to treatment with either trimethoprim-sulfamethoxazole or placebo every 2 days. By 12 months there had been no recurrences in the treatment group, while recurrence was noted in 12.8% of patients in the placebo group [43].

In conclusion, there is level I evidence that intermittent use of trimethoprim-sulfamethoxazole (every 2-3 days) following an active episode significantly reduces the risk of recurrence for at least 1 year after the active episode. Considering the low cost of this medication, use of trimethoprim-sulfamethoxazole should be strongly considered in the absence of contraindications.

## 4. Conclusions

We noted conflicting evidence as to the efficacy of systemic or intravitreous antibiotics in the treatment of* Toxoplasma* retinochoroiditis, with the preponderance of evidence suggesting that they are effective. Whilst acknowledging the limitations of the evidence available it seems that trimethoprim-sulfamethoxazole may the best first-line treatment of* Toxoplasma* retinochoroiditis, with intravitreous clindamycin with dexamethasone an alternative for patients intolerant, unresponsive or with a contraindication (such as pregnancy) to trimethoprim-sulfamethoxazole. There is level I evidence that trimethoprim-sulfamethoxazole taken intermittently reduces the risk of recurrence.

Our review did not discuss the adjuvant use of corticosteroids as this was well covered in a very recent Cochrane review that found no evidence from randomized controlled studies to support their use or indeed support concerns that their use as adjuvants to anti-Toxoplasma treatment may lead to worse outcomes [44].
